# Supplementary material for: Defining the RNA interactome by total RNA‐associated protein purification
Source: Mol Syst Biol. 2019 Apr 8;15(4):e8689. doi: 10.15252/msb.20188689 (PMC6452921; doi:10.15252/msb.20188689)
Supplement: Supplementary file 1 — Appendix [file MSB-15-e8689-s001.pdf]

Appendix to: Defining the RNA Interactome by Total RNA-Associated Protein Purification

Supplementary Text
Supplementary Figures S1 – S5

Supplementary Text

Comparison of RNP^{xl} and Xi

In order to compare the performance of Xi with previously published software tools, we analyzed our data with the RNPxl package for proteome discoverer (Veit, Sachsenberg et al., 2016). The list of possible 4-thioU fragments and other search parameters were harmonized between Xi and RNP^{xl} as much as possible and both search engines were used to analyze the same dataset (see Materials and Methods). The reported PSMs were filtered with as described in materials and methods (FDR cut-off of 1%, unique protein + crosslink site, unique peptide + crosslinked RNA). Xi performed better than RNP^{xl} with our data both in terms of the number of PSMs passing the filtering (RNP^{xl}-Xi 471-524) and in terms of the number of unique crosslinked peptides observed (RNP^{xl}-Xi 326-418). Furthermore, compared to RNP^{xl}, the proteins, identified with Xi showed a better overlap with PAR-TRAPP reported hits (Figure 6D). However, we since RNPxl does not report hit to decoy database, we had to include the decoy sequences in the search database for RNPxl, which may have negatively affected the performance.

Supplementary Text related to Figure EV6

We noted that the iTRAPP data frequently contained ions mapped to unfragmented peptide, conjugated to fragmented RNA, illustrating the problem of RNA fragmentation at the expense of peptide fragmentation. Adjusting parameters for the optimal peptide fragmentation may yield better results in the future. In addition, the figure also illustrates the limitations of the current software. The precursor in figure S6 is reported as conjugated to a 4-thiouridine, lacking an H_2S group. As expected, most of the fragmentation products are reported as conjugated to a 4-thiouridine derivative lacking the H_2S residue. However a few ions are annotated as not having the H_2S loss (b10_4tU+HPO_3, P_4tU-HPO_3, b13_4tU-HPO_3). Since it is unlikely that the indicated ions may have gained an H_2S group as a result of fragmentation, these ions should not be considered by the search engine. Thus, it would be beneficial to include in the future releases a way to specify the list of possible RNA fragments based on the conjugated nucleotide, especially so for UVC crosslinking.

Figure S1: Conserved RNA-interacting proteins
(A) Results of GO term enrichment analysis for conserved RNA interacting proteins of intermediary metabolism from yeast and bacteria, identified in Figure 4F. (B) Schematic representation of Glycolysis/Gluconeogenesis KEGG pathway identified as enriched amongst conserved RNA interacting proteins of intermediary metabolism from yeast and bacteria (Figure 4F). Proteins, present in the indicated model organism are labelled in green. Red stars indicate proteins, identified as significantly enriched in TRAPP analysis.

Figure S2: Conserved RNA-interacting proteins
Schematic representation "Purine metabolism" KEGG pathway identified as enriched amongst conserved RNA interacting proteins of intermediary metabolism from yeast and bacteria (Figure 4F). Labelling as in figure S1B.

Figure S3. Example spectrum with crosslinked alanine
MS and MS/MS spectrum of peptide, crosslinked to 4-thiouridine via an alanine residue. The observed peptide fragments are indicated on the peptide sequence with black bars. Grey bars indicate peptide fragments observed with an additional neutral loss. The software assigned site of crosslink is indicated above the peptide sequence.
Allowed RNA fragments: Ybase - 4-thiouracil base; Ybase_mH2S - 4-thiouracil base with an H2S mass loss; YmHPO3mH2S - 4-thiouridine monophosphate with HPO3 and H2S mass losses; YmH2S - 4-thiouridine monophosphate with H2S mass loss; Y - 4-thiouridine monophosphate; YmHPO3 - 4-thiouridine monophosphate with HPO3 mass loss; YpHPO3mH2S - 4-thiouridine diphosphate with an H2S mass loss; YpHPO3 - 4-thiouridine diphosphate.

Figure S4. Example spectrum with crosslinked asparagine
MS and MS/MS spectrum of peptide, crosslinked to 4-thiouridine via asparagine residue. The observed peptide fragments are indicated on the peptide sequence with black bars. Grey bars indicate peptide fragments observed with an additional neutral loss. The software assigned site of crosslink is indicated above the peptide sequence.
Allowed RNA fragments: Ybase - 4-thiouracil base; Ybase_mH2S - 4-thiouracil base with an H2S mass loss; YmHPO3mH2S - 4-thiouridine monophosphate with HPO3 and H2S mass losses; YmH2S - 4-thiouridine monophosphate with H2S mass loss; Y - 4-thiouridine monophosphate; YmHPO3 - 4-thiouridine monophosphate with HPO3 mass loss; YpHPO3mH2S - 4-thiouridine diphosphate with an H2S mass loss; YpHPO3 - 4-thiouridine

diphosphate. “P_” plus the RNA fragment denotes unfragmented peptide conjugated to an RNA fragment.

Figure S5. Example spectrum with crosslinked valine
MS and MS/MS spectrum of peptide, crosslinked to 4-thiouridine via valine residue. The observed peptide fragments are indicated on the peptide sequence with black bars. Grey bars indicate peptide fragments observed with an additional neutral loss. The software assigned site of crosslink is indicated above the peptide sequence.
Allowed RNA fragments: Ybase - 4-thiouracil base; Ybase_mH2S - 4-thiouracil base with an H2S mass loss; YmHPO3mH2S - 4-thiouridine monophosphate with HPO3 and H2S mass losses; YmH2S - 4-thiouridine monophosphate with H2S mass loss; Y - 4-thiouridine monophosphate; YmHPO3 - 4-thiouridine monophosphate with HPO3 mass loss; YpHPO3mH2S - 4-thiouridine diphosphate with an H2S mass loss; YpHPO3 - 4-thiouridine diphosphate. “P_” plus the RNA fragment denotes unfragmented peptide conjugated to an RNA fragment. “P+P” labels the unfragmented ion of Peptide conjugated to RNA. See also Supplementary Text.

A

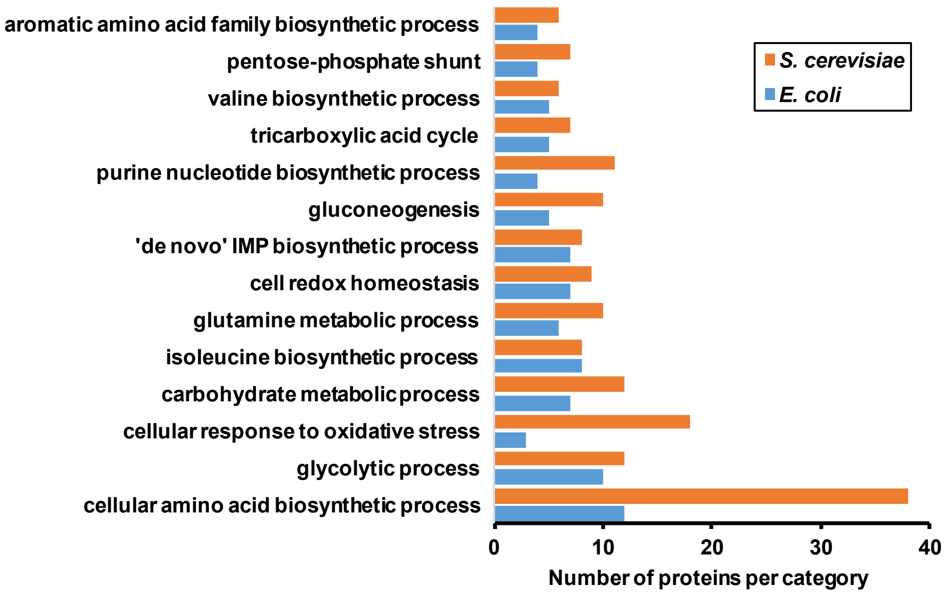

B

S. cerevisiae *E. coli*

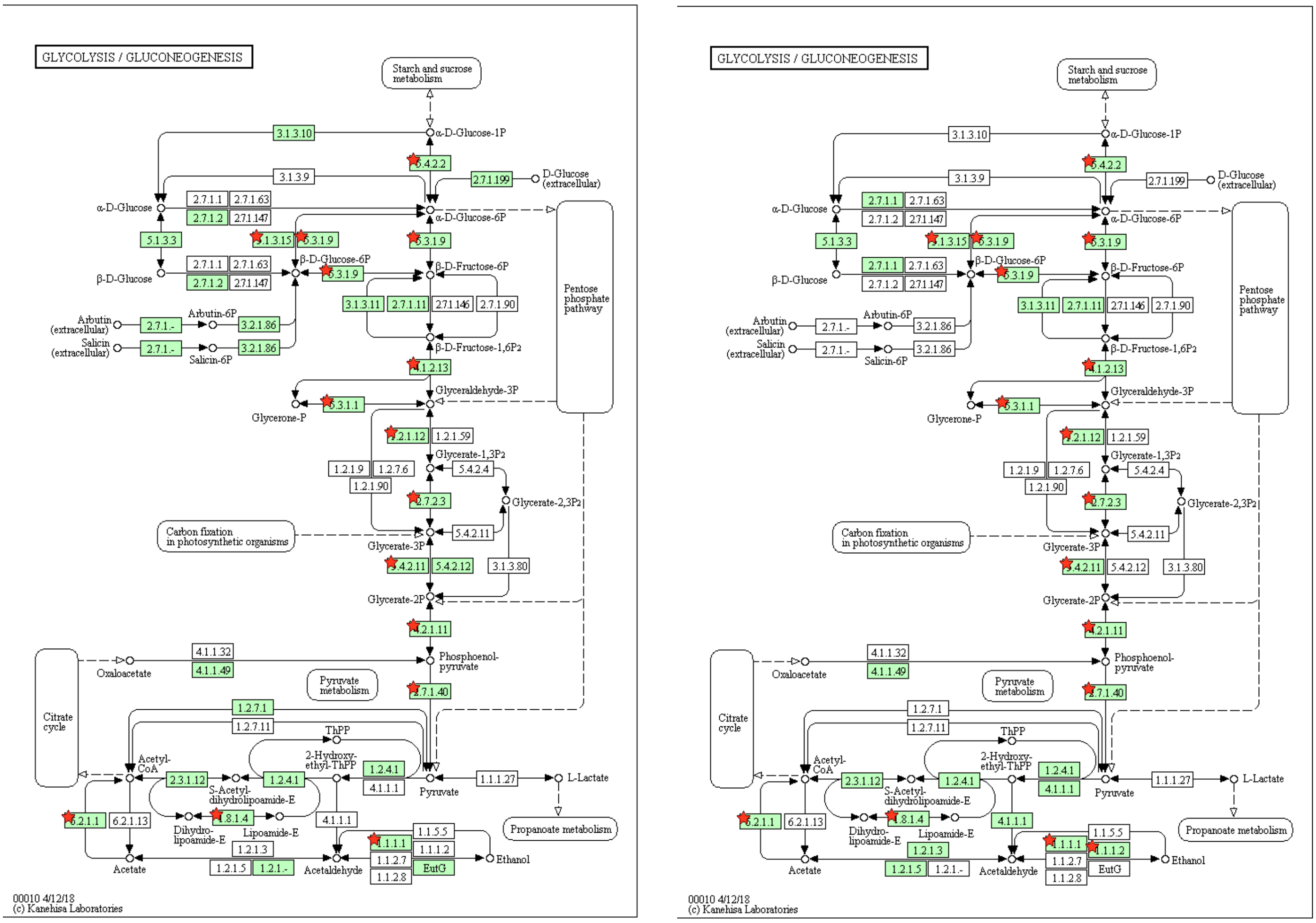

S. cerevisiae

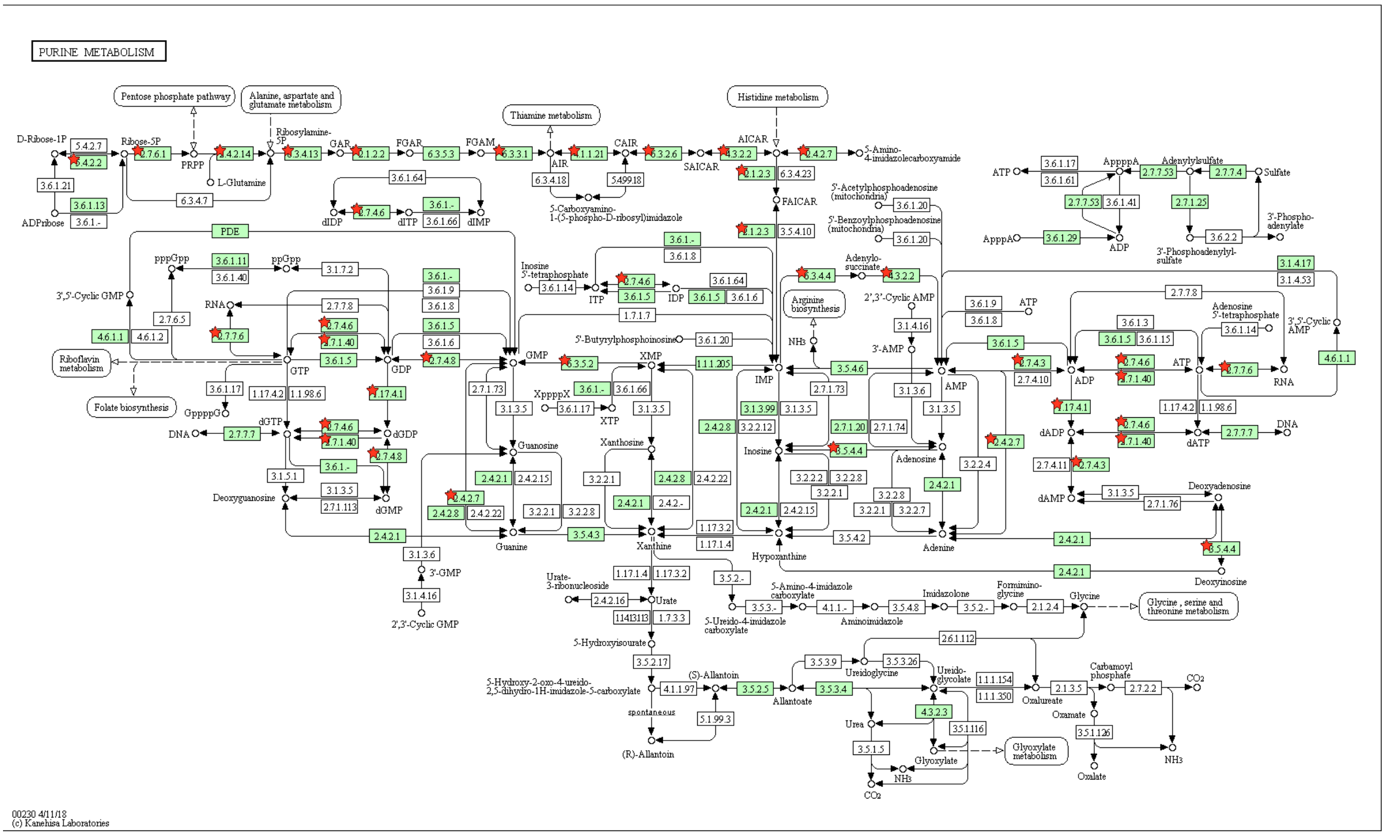

E. coli

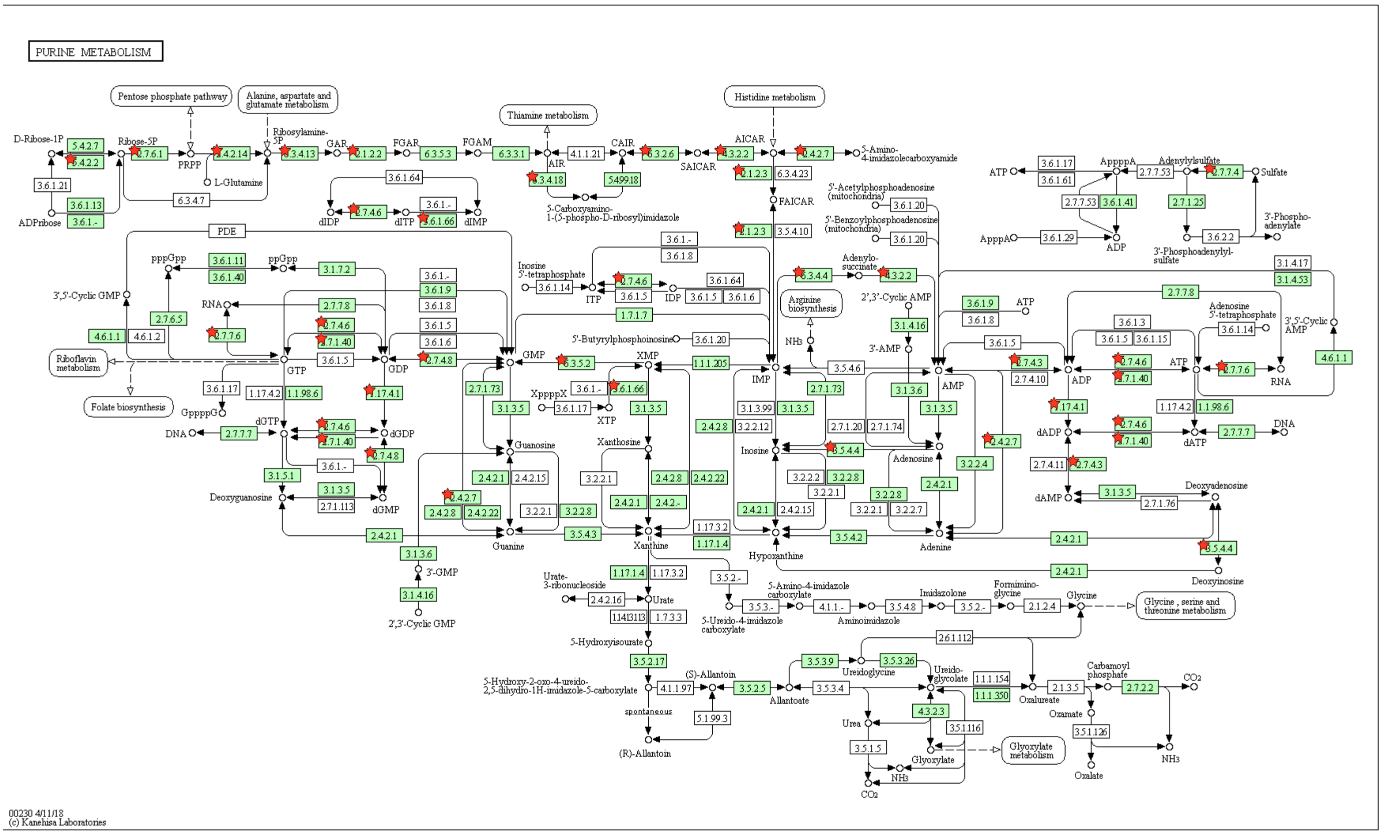

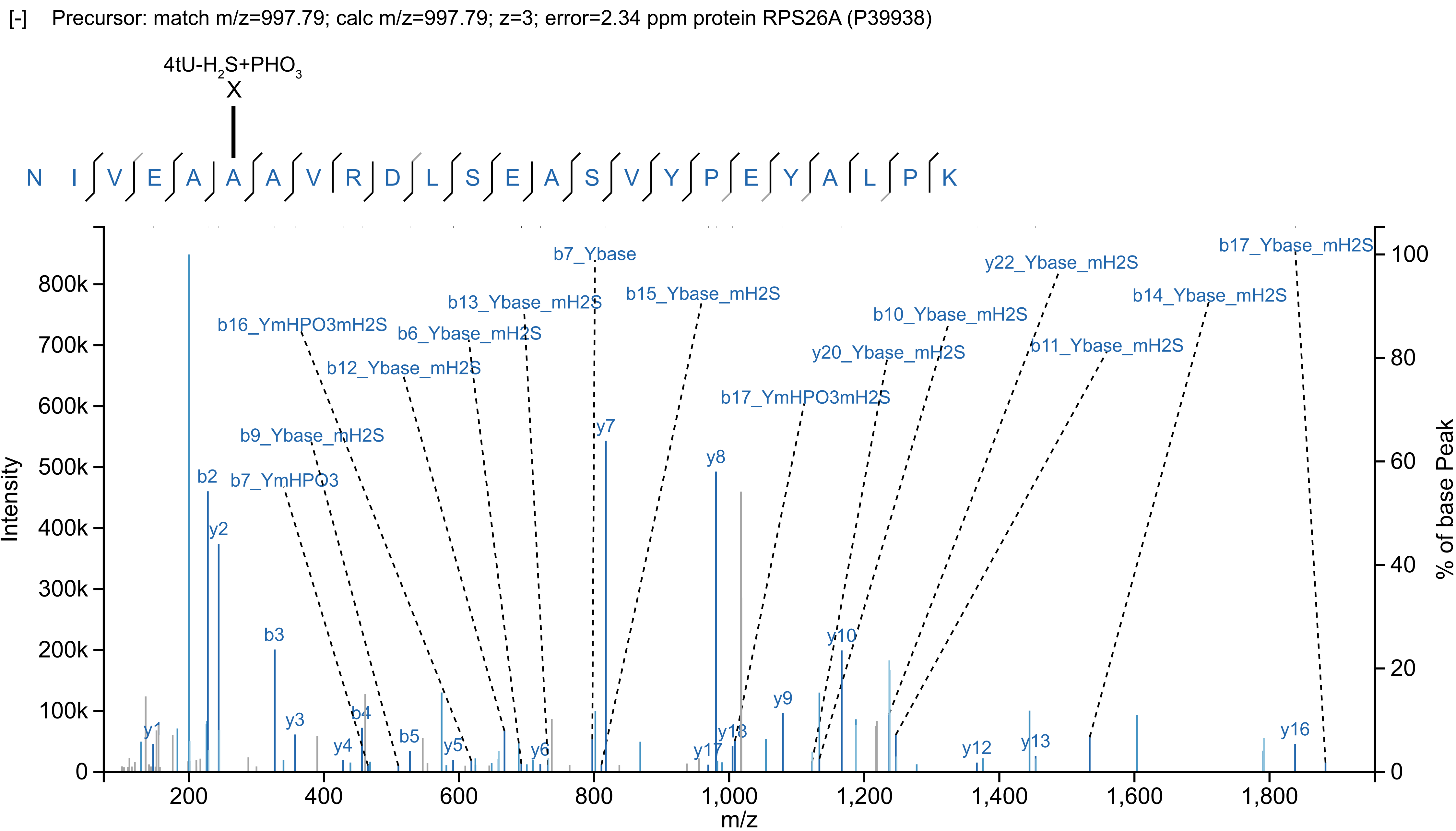

MSB-18-8689R S3

[-] Precursor: exp m/z=818.01; calc m/z=818.01; z=3; error=1.09 ppm; Protein NUG1 (P40010)

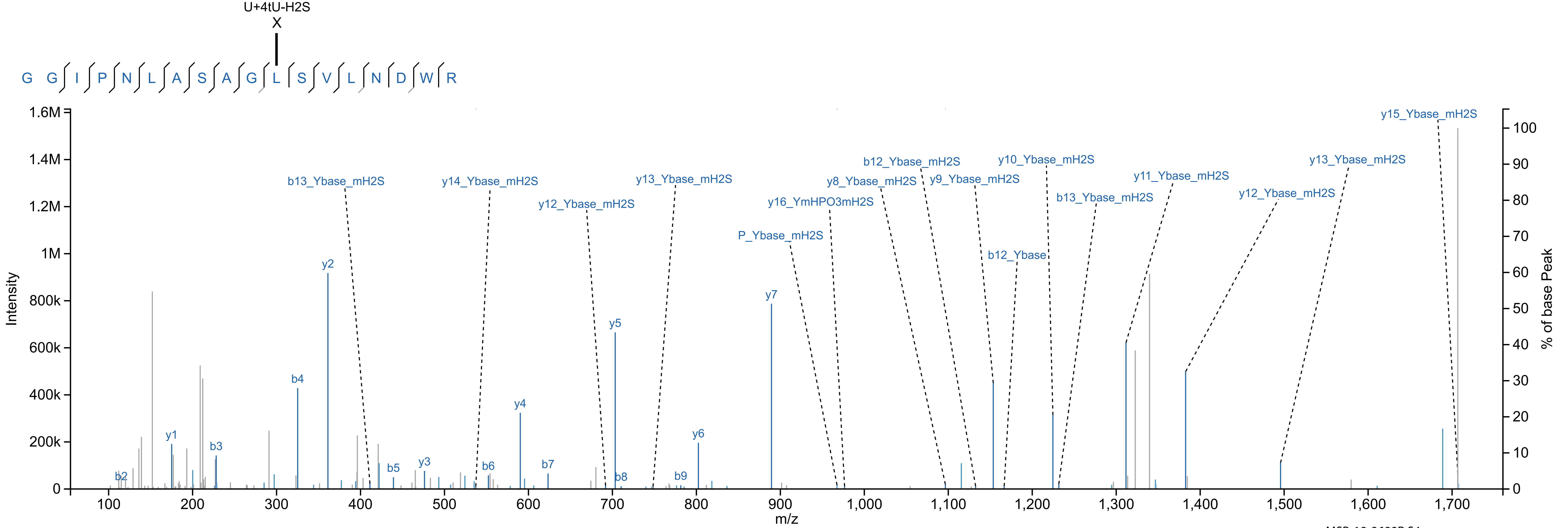

MSB-18-8689R S4

[-] Precursor: match m/z=667.05; calc m/z=667.05; z=4; error=2.04 ppm; RPS8A;RPS8B (P0CX39;P0CX40)

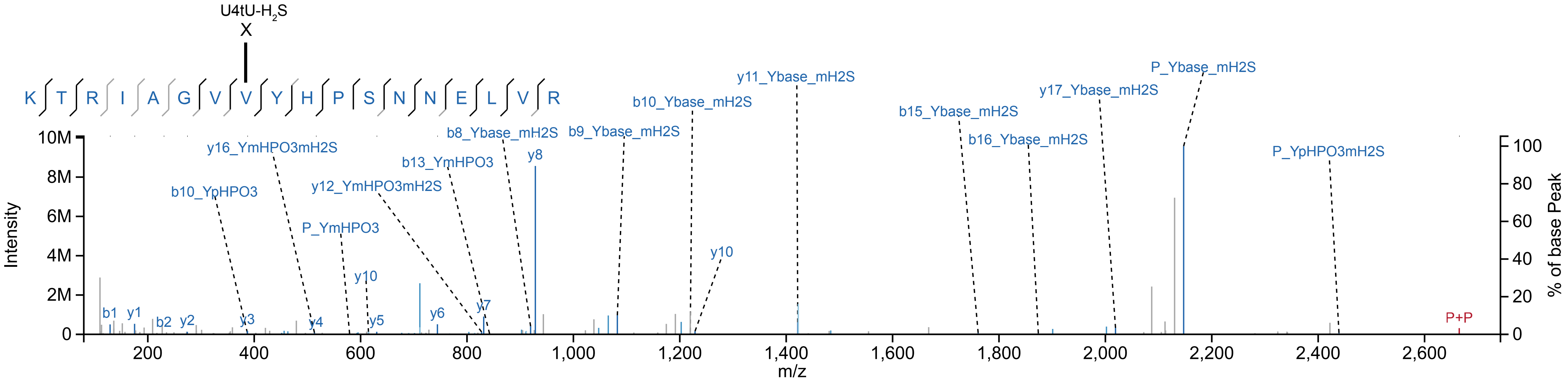

MSB-18-8689R S5
