# Supplementary material for: Defining the RNA interactome by total RNA‐associated protein purification
Source: Mol Syst Biol. 2019 Apr 8;15(4):e8689. doi: 10.15252/msb.20188689 (PMC6452921; doi:10.15252/msb.20188689)
Supplement: Supplementary file 2 — Expanded View Figures PDF [file MSB-15-e8689-s002.pdf]

Expanded View Figures

Figure EV1. TRAPP and PAR-TRAPP techniques and reproducibility.

A Pairwise Pearson correlation coefficients for protein Log_2 +UV/−UV ratios obtained in *S. cerevisiae* with TRAPP and PAR-TRAPP experiments. Forward isotopic labelling and reverse isotopic labelling are indicated as "FWD" and "RV", respectively.

B Data for *Escherichia coli* 1 forward and 2 reverse labelling TRAPP repeats were processed as in (A).

C The analysis of *S. cerevisiae* forward and reverse PAR-TRAPP experiments upon sorbic acid exposure performed as in (A).

D Effect of 4tU and UVA treatments on the growth of yeast cells. Exponentially growing yeast cells were treated for 2 h with 4-thiouracil at the indicated concentrations. The cultures were then irradiated with 350 nm UVA light in the eBox for 30 s delivering 5.8 J cm^{-2}. The lag time of treated cultures was measured by monitoring samples growth curve with Tecan sunrise instrument. Samples: "4tU wash out +UVA"—growth delay of 4-thiouracil-treated UVA-irradiated cells, compared to UVA-exposed sample. 4tU was removed prior to irradiation; "4tU w/o wash out +UVA"—As sample 1, but 4tU persisted in the media while cells were irradiated; "4tU alone"—growth delay of cells treated with 4tU for 2 h as compared to untreated cells without irradiation.

E Frontal view on the eBox irradiation apparatus. The frontal door and the shutters are not present on the picture. Red arrows indicate rails for shutters, designed to prevent sample exposure to UV light, while the lamps are warming up for stable UVA output. The UVA transparent sample tray made of borosilicate glass is placed between the two UVA lamp banks.

© 2019 The Authors

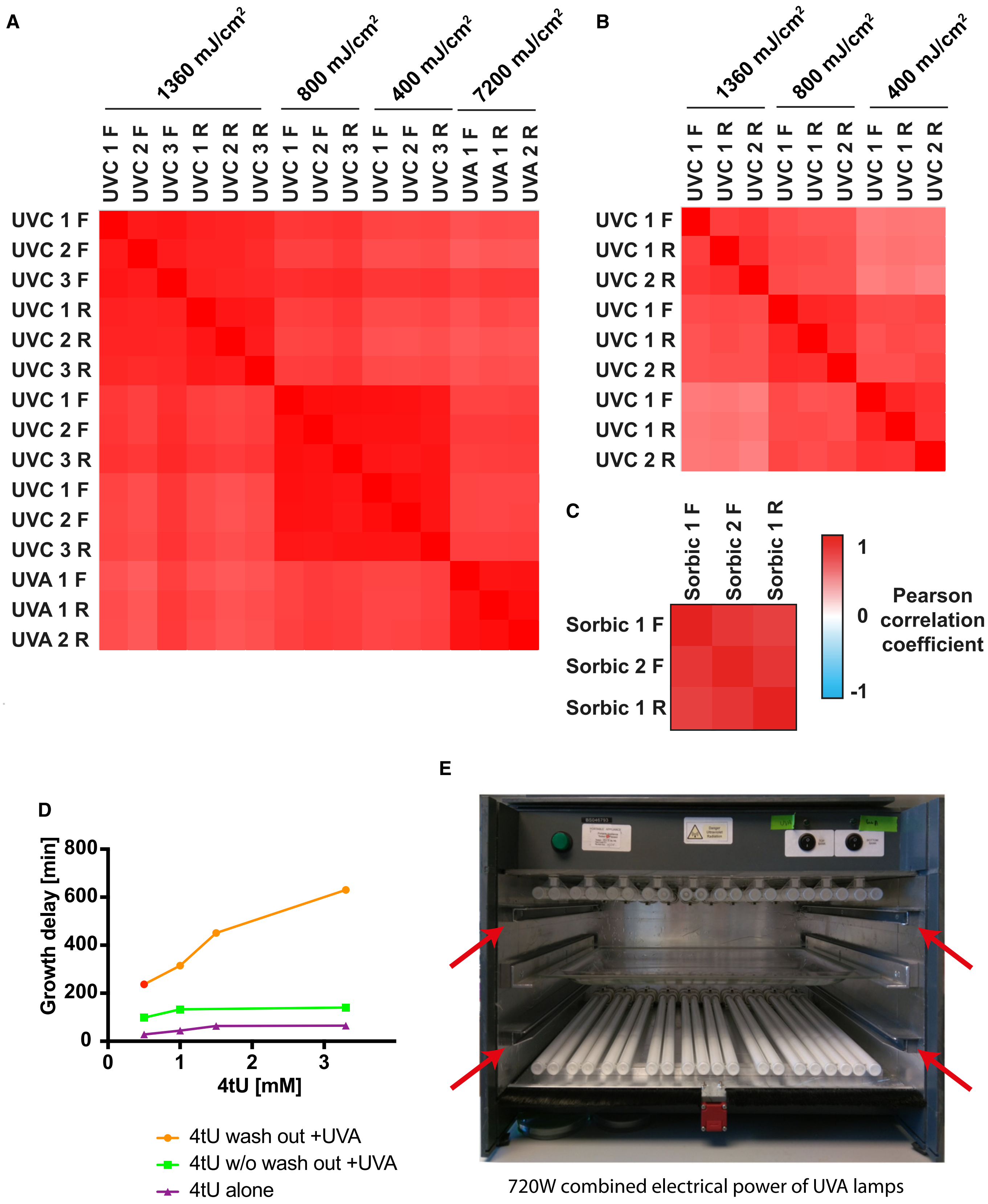
A
1360 mJ/cm²
800 mJ/cm²
400 mJ/cm²
7200 mJ/cm²
UVC 1 F
UVC 2 F
UVC 3 F
UVC 1 R
UVC 2 R
UVC 3 R
UVC 1 F
UVC 2 F
UVC 3 R
UVC 1 F
UVC 2 F
UVC 3 R
UVA 1 F
UVA 1 R
UVA 2 R
B
1360 mJ/cm²
800 mJ/cm²
400 mJ/cm²
UVC 1 F
UVC 1 R
UVC 2 R
UVC 1 F
UVC 1 R
UVC 2 R
UVC 1 F
UVC 1 R
UVC 2 R
C
Sorbic 1 F
Sorbic 2 F
Sorbic 1 R
1
0
-1
Pearson correlation coefficient
D
Growth delay [min]
800
600
400
200
0
0
1
2
3
4tU [mM]
4tU wash out +UVA
4tU w/o wash out +UVA
4tU alone
E
720W combined electrical power of UVA lamps

Figure EV1.

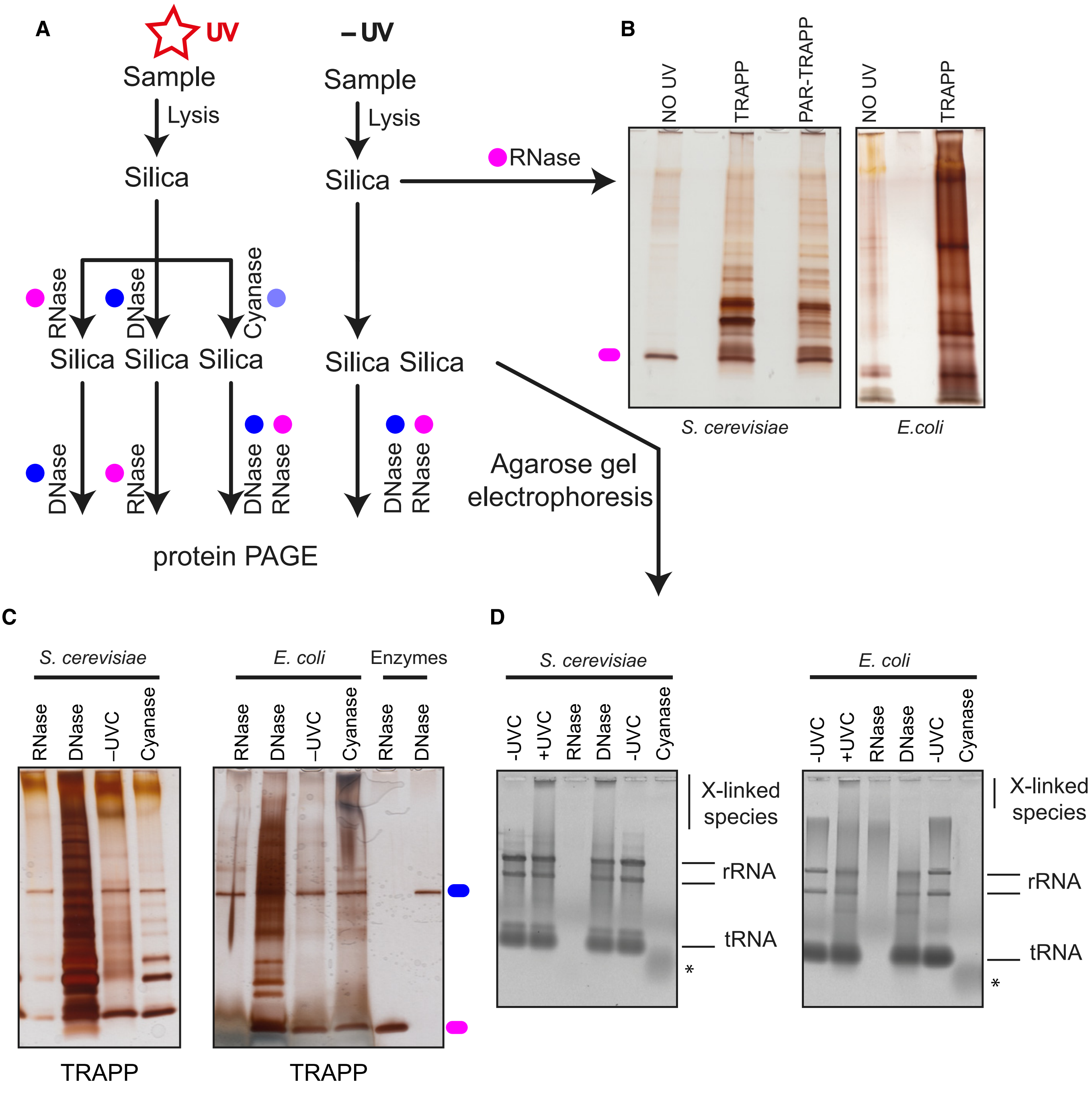

Figure EV2. TRAPP protocol predominately recovers RNA-bound proteins.

A The experimental set-up indicating the stages when samples are collected. Coloured circles designate treatment with the indicated enzyme.

B Samples are purified following the TRAPP protocol as described in Materials and Methods. After RNase A and RNase T1 treatment to degrade the co-purifying RNA, sample was resolved on polyacrylamide gel and silver staining was performed.

C TRAPP-purified samples were treated with the indicated enzymes and loaded onto silica once again. After elution, nucleic acids were resolved with agarose gel electrophoresis (see Fig EV2D), while the remainder of the sample was treated with the indicated enzyme followed by polyacrylamide gel electrophoresis and silver staining.

D Same as in (C), but the samples were collected before the second nuclease treatment and were then resolved on a SYBR Safe stained agarose gel. * denotes residual nucleic acid species in the cyanase-treated sample.

 © 2019 The Authors

Figure EV3. Quantification of TRAPP and PAR-TRAPP data.

A The percentage of peptides with reported intensity in +UV sample, but not in −UV sample (superenriched peptides) by MaxQuant in *Saccharomyces cerevisiae* TRAPP (at 1,360 mJ cm^{-2}) SILAC quantification experiments without (black bars) or with (grey bars) "requantify" option enabled. 3 biological repeats had light-labelled cells UV irradiated (1F, 2F,3F), while three other repeats (1R, 2R, 3R) had heavy-labelled cells UV irradiated.

B The data of *S. cerevisiae* PAR-TRAPP experiments were analysed the same way as in (A).

C The data of *E. coli* TRAPP experiments were analysed the same way as in (A), except the 2 biological repeats, which had light-labelled cells UV irradiated, were labelled 1R and 2R.

D The percentage of peptides with reported intensity in −UV sample, but not in +UV sample (superdepleted peptides) by MaxQuant in *S. cerevisiae* TRAPP (at 1,360 mJ cm^{-2}) without (dotted) or with (chequered) "requantify" option enabled. Sample labelling as in (A).

E The data of *S. cerevisiae* PAR-TRAPP experiments were analysed the same way as in (D).

F The data of *E. coli* TRAPP (at 1,360 mJ cm^{-2}) experiments were analysed the same way as in (D), sample labelling was as in panel (C).

G Box plot of Log_{10} peptide intensity of −UV peptides from *S. cerevisiae* TRAPP (at 1,360 mJ cm^{-2}) (blue) samples (labelling as in (A)), plotted together with Log_{10} peptide intensity values imputed by imputeLCMD R package for −UV samples (orange). Box represents values between 25th and 75th percentiles, while whiskers represent 10th and 90th percentiles. All other data are represented as points below or above 10th or 90th percentiles, respectively. Line inside the box shows median value.

H Histogram of peptide intensity frequency obtained from −UV sample (1F), plotted for intensities from 0 to 5×10^5 units. Colour labelling is as in (G).

I Same as panel (H), performed for sample 1R which had reversed SILAC labelling, compared to the sample analysed in panel (H).

J Box plot of Log_{10} peptide intensity of −UV peptides from *S. cerevisiae* PAR-TRAPP (blue) samples (labelling as in (B)), plotted together with Log_{10} peptide intensity values imputed by imputeLCMD R package for −UV samples (orange).

K Histogram of peptide intensity frequency obtained from −UV sample (1F), plotted for intensities from 0 to 5×10^5 units. Colour labelling is as in (J).

L Same analysis as panel (K), performed for sample 1R which had reversed SILAC labelling, compared to the sample analysed in panel (K).

M Box plot of Log_{10} peptide intensity of −UV peptides from *E. coli* TRAPP (at 1,360 mJ cm^{-2}) (blue) samples (labelling as in (C)), plotted together with Log_{10} peptide intensity values imputed by imputeLCMD R package for −UV samples (orange).

N Histogram of peptide intensity frequency obtained from −UV sample (1F), plotted for intensities from 0 to 5×10^5 units. Colour labelling is as in (M).

O Same analysis as panel (N), performed for sample 1R which had reversed SILAC labelling, compared to the sample analysed in panel (N).

© 2019 The Authors

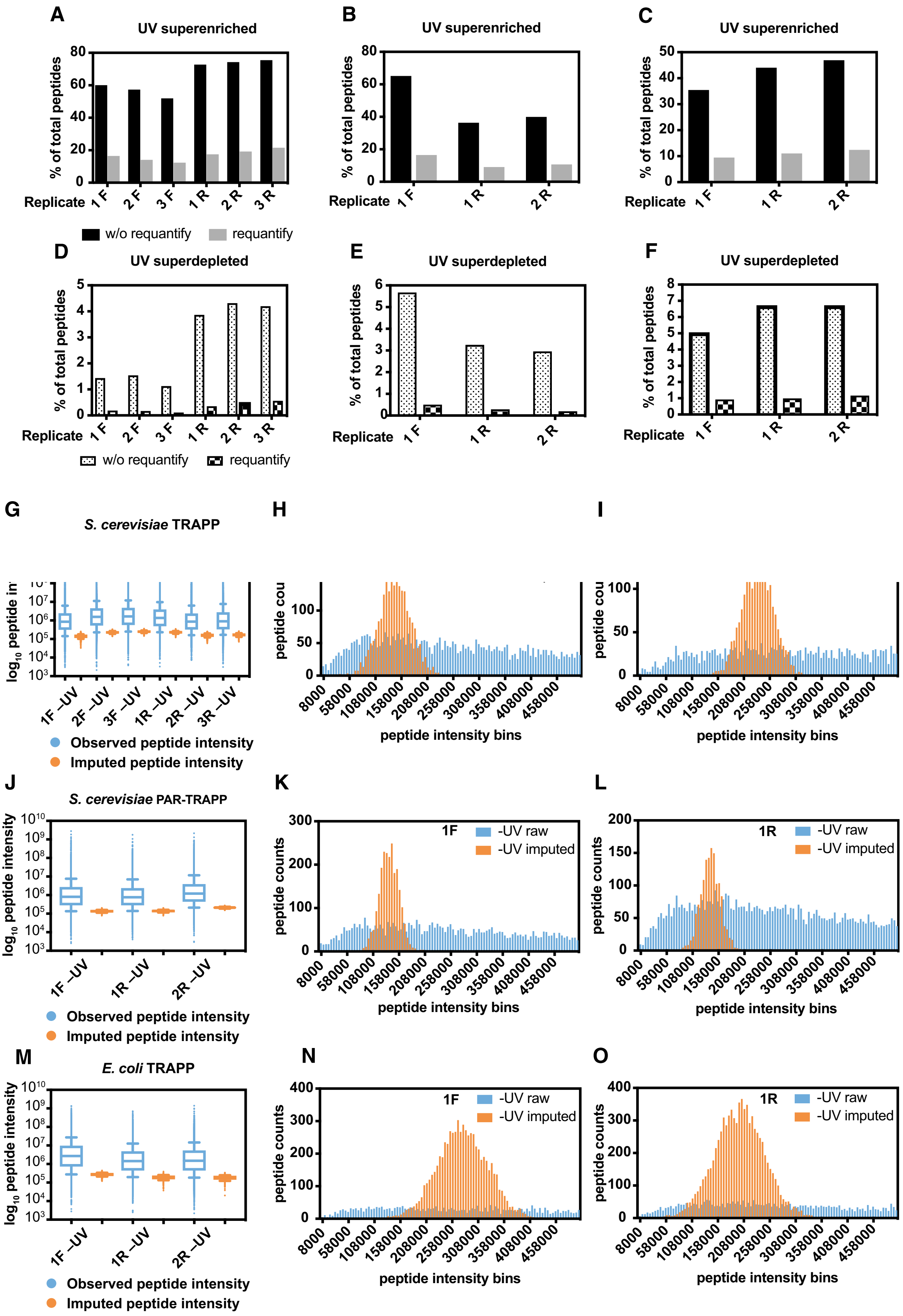

Figure EV3.

© 2019 The Authors

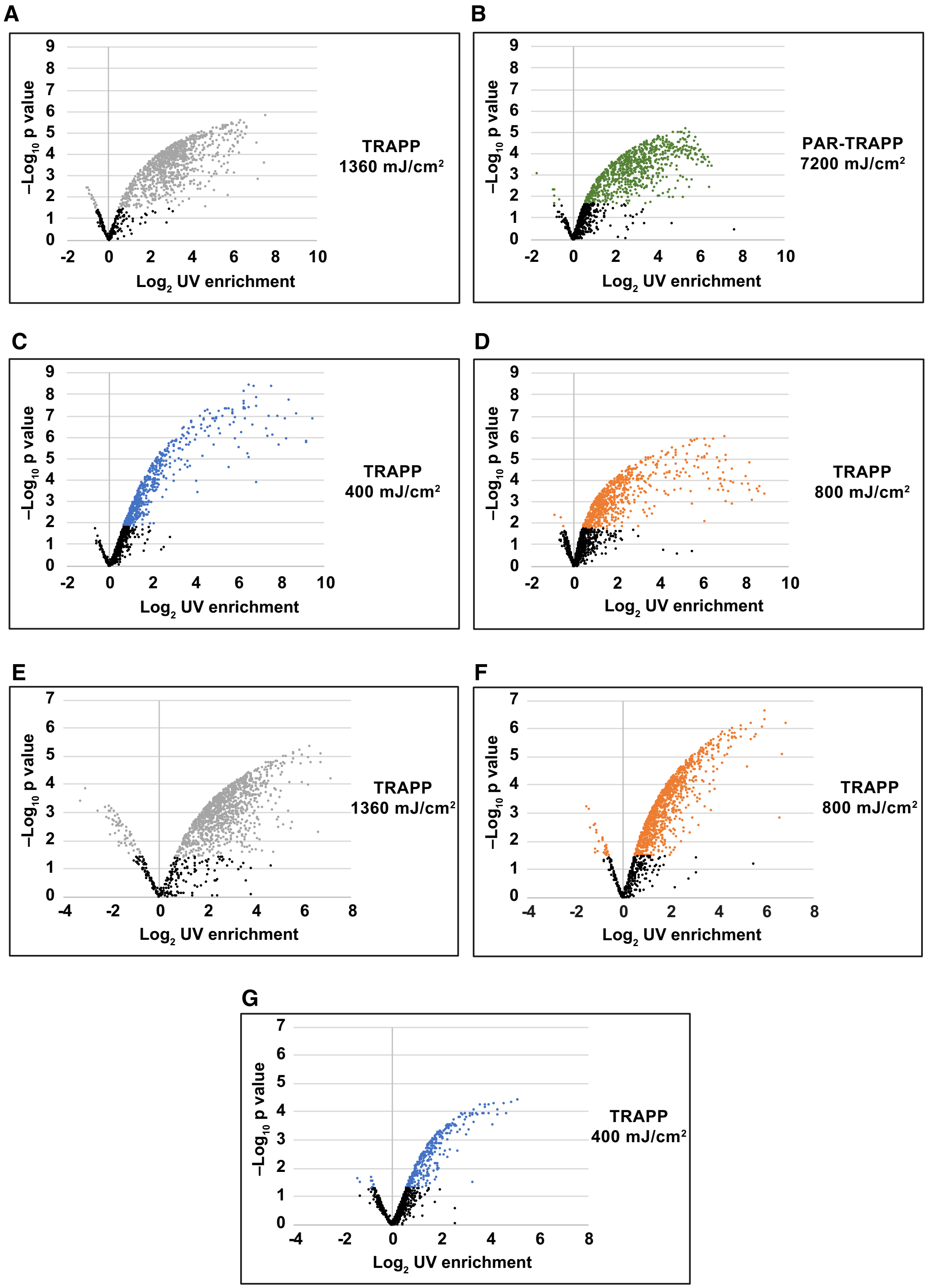

Figure EV4. Volcano plots for protein enrichment in TRAPP with different UV exposure.

A–G Volcano plot showing Log_2 UV fold enrichment plotted against $-Log_{10}$ per protein for the following experiments: (A) *Saccharomyces cerevisiae* TRAPP at 1,360 mJ cm^{-2}; (B) *S. cerevisiae* PAR-TRAPP at 7.2 J cm^{-2}; (C) *S. cerevisiae* TRAPP at 400 mJ cm^{-2}; (D) *S. cerevisiae* TRAPP at 800 mJ cm^{-2}; (E) *E. coli* TRAPP at 1,360 mJ cm^{-2}; (F) *E. coli* TRAPP at 800 mJ cm^{-2}; (G) *E. coli* TRAPP at 400 mJ cm^{-2}.

© 2019 The Authors

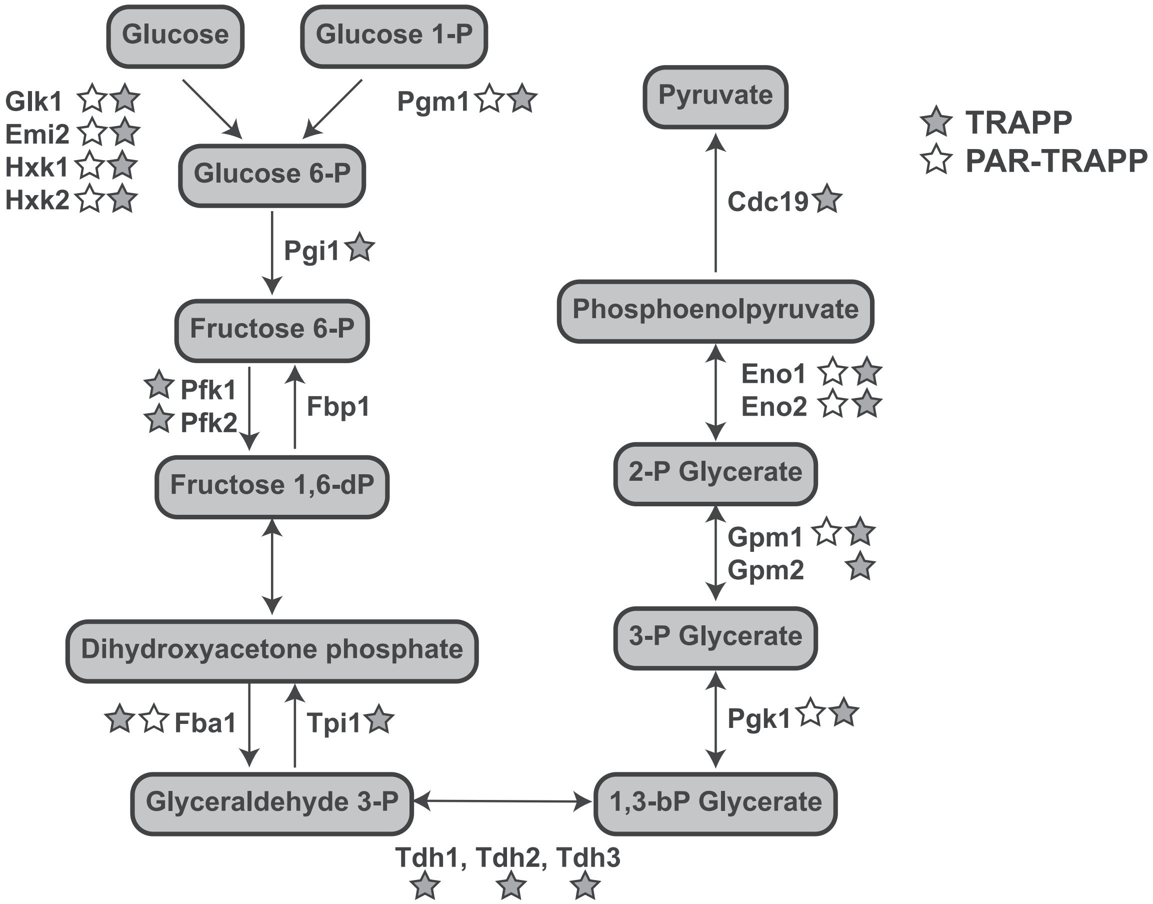

Figure EV5. Glycolytic enzymes identified in TRAPP.

Glycolysis pathway in yeast *Saccharomyces cerevisiae*, indicating intermediate metabolites and participating enzymes. Proteins identified as enriched by TRAPP (1.4 J cm^{-2}) and PAR-TRAPP are shown with grey and white stars, respectively.

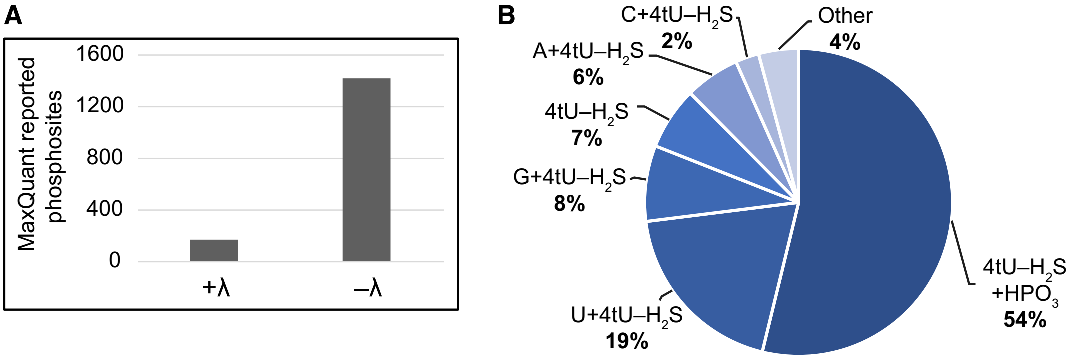

Figure EV6. iTRAPP including Lambda phosphatase treatment.

A The number of protein phosphorylation sites, reported by MaxQuant software for the λ phosphatase-treated sample and untreated control, demonstrating that the treatment effectively removed phosphorylation from amino acids.

B Pie chart of RNA species observed crosslinked to peptides by the Xi search engine in the sample treated with Lambda phosphatase.

© 2019 The Authors
